# Real‐World Evaluation of the BioFire FilmArray ME Panel: Diagnostic Accuracy and Clinical Utility

**DOI:** 10.1155/cjid/3714674

**Published:** 2026-03-06

**Authors:** Mehmet Erinmez, Mustafa Sağlam, Gönenç Çalışkantürk, Merve Özerol, Yasemin Zer

**Affiliations:** ^1^ Department of Medical Microbiology, Gaziantep University Faculty of Medicine, Gaziantep, Türkiye, gantep.edu.tr; ^2^ Gaziantep Provincial Directorate of Health Nizip District State Hospital, Gaziantep, Türkiye

## Abstract

Central nervous system (CNS) infections carry high morbidity and mortality, yet traditional diagnostics often fail to identify the causative agent promptly. Rapid multiplex panels offer broader and faster pathogen detection, making it important to understand their real‐world clinical value. In order to evaluate the diagnostic performance of the multiplex panel, panel results were compared with clinical, laboratory, and imaging data. Concordance between the panel and reference methods defined true‐positive and true‐negative results, and discrepancies were classified as false positives or false negatives. A total of 955 patients were included. The FilmArray ME panel detected at least one pathogen in 8.4% of patients. *Streptococcus pneumoniae* was the most common bacterial agent, and *Enterovirus* and HSV‐1 were the most frequent viral detections. Overall sensitivity and specificity were 94.8% and 99.4%. Sensitivity was 91.6% in children and 97.6% in adults, and specificity was 99.6% in children and 99.1% in adults. We also identified in 3.9% of cases bacterial pathogens outside the ME panel’s coverage, most commonly *Acinetobacter baumannii* and *Stenotrophomonas maltophilia*, organisms usually associated with healthcare‐related or neurosurgical infections. Our large real‐world cohort shows that the FilmArray ME panel provides rapid, accurate detection of key meningitis and encephalitis pathogens. Nonetheless, false‐negative bacterial findings, detection of viruses with limited clinical relevance, and the presence of infections due to organisms outside the panel indicate that it should complement, not replace, conventional diagnostics and careful clinical assessment.

## 1. Introduction

Meningitis and encephalitis represent separate yet closely interconnected infectious processes involving the central nervous system (CNS) [1]. Meningitis refers to the inflammation of the meninges, whereas encephalitis denotes inflammation of the brain parenchyma. Meningoencephalitis describes a condition in which both the meninges and the brain parenchyma are concurrently inflamed [2]. The clinical manifestations of these conditions are often not distinctive. Meningitis commonly presents with systemic features including fever, photophobia, headache, and stiffness of the neck, whereas encephalitis is more frequently marked by disturbances in consciousness or cognition [1]. Meningitis and encephalitis may arise from viral, bacterial, or fungal infections, although encephalitis is more frequently linked to viral pathogens [2]. Infectious meningitis and encephalitis are neurologic disorders capable of producing severe clinical outcomes. These illnesses are associated with significant morbidity and mortality, with bacterial meningitis posing an especially urgent threat due to its potential for rapid progression and fatality [3]. Individuals who survive these infections may experience considerable long‐term complications, including amputation, visual and auditory deficits, seizure disorders, and impairments in cognitive function [4]. Moreover, these infections impose substantial economic burdens, encompassing immediate costs such as hospitalization and medical management, as well as long‐term consequences including reduced productivity and broader societal impact [5].

While empirical therapy is frequently employed, accurately identifying the causative agent and promptly initiating targeted treatment are critical for optimizing patient outcomes, with the greatest clinical benefit observed when appropriate antimicrobial therapy is administered without delay [2, 3]. Nonetheless, the diagnosis of meningitis and encephalitis is fraught with challenges. Clinical manifestations are often heterogeneous, and both systemic symptoms and focal neurological signs frequently overlap across different infectious etiologies, complicating accurate identification [6]. Confirming an infectious etiology is often difficult and time‐consuming [7]. Although cerebrospinal fluid and peripheral blood cultures are routinely used in the diagnostic evaluation, their turnaround time is lengthy, often requiring several days. Moreover, depending on the clinical context, negative results do not reliably exclude infection [1]. Routine testing such as cellular (white blood cell [WBC] and erythrocyte counts) and biochemical parameters (protein and glucose concentrations) in the CSF may provide clues regarding the nature of the infection (bacterial, viral, or fungal), yet these parameters are not definitive [8]. A considerable proportion of meningitis and encephalitis cases, particularly those attributable to viral pathogens, may evade definitive diagnosis despite clinical evaluation [9]. The underlying cause of meningitis or encephalitis is established in only a minority of patients, typically around one‐third to one‐half of cases, a limitation largely attributable to the inadequacy of current diagnostic approaches [10]. Clinicians must often make consequential therapeutic decisions, including whether to initiate antibacterial therapy, antiviral therapy, a combination of both, or to opt for supportive management when the presentation suggests a self‐limiting viral aseptic meningitis or a noninfectious process. In some situations, the consideration of corticosteroid use further complicates this decision‐making [1]. Prolonged turnaround times for diagnostic tests, low rates of pathogen detection, and the challenge of distinguishing incompletely managed bacterial meningitis from viral meningitis can contribute to superfluously extended courses of antibiotics and prolonged hospital stays [11].

Syndromic panel assays represent an emerging multiplex molecular approach that has gained widespread adoption for the diagnostic evaluation of infections involving the bloodstream, respiratory system, gastrointestinal tract, and the CNS [2]. These multiplex assays represent a transformative advancement, allowing clinicians to rapidly identify infectious agents and thereby make timely decisions regarding patient management, including hospitalization, isolation measures, and the initiation or discontinuation of antimicrobial therapy [2]. This approach allows for the identification of pathogens within hours, markedly faster than traditional methods that generally take 2 to 3 days, thereby facilitating the earlier administration of targeted antimicrobial therapy, a consideration of particular importance in CNS infections given their significant mortality risk [12]. The FilmArray Meningitis and Encephalitis Panel (MEP) (BioFire Diagnostics/BioMérieux, Salt Lake City, UT) enables rapid, simultaneous PCR‐based detection of 14 targets: 6 bacteria (*Escherichia coli* K1, *Haemophilus influenzae*, *Listeria monocytogenes*, *Neisseria meningitidis*, *Streptococcus agalactiae*, and *Streptococcus pneumoniae*), 7 viruses (cytomegalovirus [CMV], *Enterovirus*, Herpes simplex virus 1/2 (HSV‐1 and HSV‐2), human herpesvirus 6 [HHV‐6], Human parechovirus, and Varicella‐zoster virus [VZV]), and one yeast (*Cryptococcus neoformans*/*gattii*) capable of causing CNS infections [9].

Timely and precise identification of pathogens in cerebrospinal fluid can enhance the clinical management of patients with suspected CNS infections [13]. Although these assays deliver prompt results that can meaningfully guide clinical decision‐making, inaccurate findings or misinterpretation by clinicians may lead to inappropriate management and potentially harm patients. In the end, the laboratory must balance the dual responsibility of maintaining analytical accuracy while also guaranteeing that the findings it reports are clinically meaningful and actionable [1]. Nonetheless, the real‐world effectiveness of the MEP relative to conventional diagnostic approaches is not fully characterized, nor is its potential influence on routine clinical practice [14]. In this study, we aim to systematically analyze the clinical and laboratory findings and evaluate the diagnostic accuracy of the ME panel, as well as its impact on the management and clinical outcomes of CNS infections.

## 2. Materials and Methods

### 2.1. Clinical Setting and Patient Data

This study was conducted at Şahinbey Research and Application Hospital, a tertiary university hospital located in southeastern Türkiye. The study comprised an ambidirectional cohort covering the period from February 2018 to October 2025, corresponding to the timeframe surrounding the introduction of the multiplex assay (The FilmArray; BioFire Diagnostics/BioMérieux). Patients were included in the study if they had undergone a lumbar puncture as part of the assessment for a potential CNS infection. The BioFire ME panel was requested as a syndromic test for patients with suspected CNS infection, in line with routine clinical practice. As a multiplex system designed by the manufacturer, the panel simultaneously detects a predefined set of bacterial, viral, and fungal pathogens, regardless of the level of clinical suspicion for each individual pathogen. Data extracted from medical records encompassed patient demographics (age and sex), details of antibiotic therapy and its duration, laboratory results including blood WBC count, blood and urine cultures, and CSF analysis (WBC count, glucose, and protein) along with CSF culture. In addition, findings from brain imaging were documented.

### 2.2. Sample Processing and Definitions

Standard procedures for CSF analysis, including cell count, Gram staining, culture, and biochemical testing, were performed for all specimens. Reference methods used for performance comparison of the multiplex assay were predefined according to the pathogen group. For bacterial and fungal pathogens, CSF culture was accepted as the reference standard [9]. Bacterial and fungal cultures were performed on every specimen enrolled. CSF samples were inoculated onto Columbia Agar with 5% sheep blood (BD, USA), Eosin Methylene Blue agar (EMB) (BD, USA), Chocolate agar (BD, USA), and Sabouraud Dextrose Agar (SDA) (BD, USA) and were incubated at 35°C–37°C in 5% CO2 for 2–5 days.

Abnormal CSF biochemical findings [15] (glucose ≤ 70 mg/dL or less than two‐thirds of the concurrently measured blood glucose level, protein ≥ 50 mg/dL), abnormal CSF cytology (leukocyte count ≥ 5 cells/μL with a predominance of polymorphonuclear leukocytes or, in the case of fungal etiology, absence of leukocytes or lymphocytic predominance), clinical features suggestive of bacterial or fungal infection (such as fever, altered mental status, meningeal irritation signs, headache, rash, seizures, photophobia, or focal neurological deficits), pathogen‐directed antimicrobial or antifungal therapy, elevated C‐reactive protein (CRP) levels, and supportive radiological findings (including leptomeningeal enhancement or echogenic alterations in the brain parenchyma) were collectively considered supportive diagnostic evidence. For viral infections, the reference classification relied on a composite assessment of abnormal CSF biochemistry (glucose ≥ 70 mg/dL or more than two‐thirds of the concurrently measured blood glucose level, protein ≥ 50 mg/dL), CSF cytology (leukocyte count ≥ 5 cells/μL with lymphomonocytic predominance), clinical features consistent with viral infection, pathogen‐directed antiviral therapy, elevated CRP levels, and supportive radiological findings.

An independent chart review of discordant patient results was conducted by two investigators with expertise in neurology (Çalışkantürk, G. and Zengin, F). True‐positive (TP) and true‐negative (TN) results were defined as those in which the FilmArray ME panel and reference methods were concordant. Conversely, false‐positive (FP) and false‐negative (FN) results were defined as those showing discordance between the index test and the reference method.

### 2.3. Calculations and Statistical Analysis

Sensitivity was calculated as 100 × (TP/[TP + FN]), and specificity was calculated as 100 × (TN/[TN + FP]). Positive predictive value (PPV) was defined as the proportion of TP results among all positive test results and was calculated using the formula TP/(TP + FP). Two‐sided 95% confidence intervals (CIs) for both performance metrics were computed using the exact binomial method based on the Wilson score method [14].

## 3. Results

A total of 955 unique patients were evaluated, of whom 35.6% were pediatric and 64.4% were adults. The cohort comprised 55.3% males and 44.7% females, with a mean age of 34.6 years. According to the FilmArray ME panel, the overall positivity rate among patients was approximately 8.4%. The most common bacterial pathogen was *S. pneumoniae* (28.7% of positive cases), followed by *H. influenzae* (7.5%) and *N. meningitidis* (6.2%). Also, in a single patient, both *H. influenzae* and *L. monocytogenes* were detected concurrently. Viral pathogens accounted for a substantial proportion, with *Enterovirus* (15%) and HSV‐1 (13.7%) being the most frequent. Less common pathogens included *E. coli*, *L. monocytogenes*, VZV, and *C. neoformans/gattii* (Table 1).

**TABLE 1:** Microorganisms detected by the FilmArray ME panel.

Microorganism	*n* (%)
*Streptococcus pneumoniae*	23 (28.7)
*Haemophilus influenzae*	6 (7.5)
*Neisseria meningitidis*	5 (6.2)
*Escherichia coli*	3 (3.7)
*Listeria monocytogenes*	1 (1.2)
*Streptococcus agalactiae*	0 (0)
*Enterovirus*	12 (15)
Herpes simplex virus 1	11 (13.7)
Human herpesvirus 6	8 (10)
Cytomegalovirus	6 (7.5)
Varicella‐zoster virus	3 (3.7)
Herpes simplex virus 2	0 (0)
Parechovirus	0 (0)
*Cryptococcus neoformans/gattii*	2 (2.5)

Overall test performance was high, with a sensitivity of 94.8% and a specificity of 99.4%. In pediatric patients, sensitivity and specificity were 91.6% and 99.6%, respectively, whereas in adults, sensitivity increased to 97.6% with a specificity of 99.1% (Table 2). For bacterial pathogens, the PPV of the test was 97.3%, based on 36 TP and 1 FP results. For viral agents, the PPV of the test was 90.0%, based on 36 TP and 4 FP results. For fungal agents, the PPV of the test was 100% (2 TPs and no FPs); however, this estimate is based on a very limited number of positive cases.

**TABLE 2:** Test performance analysis by general, pediatric, and adult populations.

Metric	General		Pediatric		Adult	
	Value (%)	95% CI^∗^	Value (%)	95% CI^∗^	Value (%)	95% CI^∗^
Sensitivity	94.8%	87.5–97.9	91.6	78.1–97.1	97.6	87.6–99.5
Specificity	99.4%	98.6–99.7	99.6	98.1–99.9	99.1	97.9–99.6

^∗^CI, confidence interval.

Among the four FN results, all patients exhibited clinical and laboratory findings compatible with meningitis; cerebrospinal fluid cultures yielded *E. coli* in two patients and *S. pneumoniae* in the remaining two patients, although these pathogens are included in the meningitis encephalitis multiplex panel, and all corresponding FilmArray ME panel results were reported as negative (Table 3). Among the FN cases with CSF cultures positive for *S. pneumoniae*, one adult patient had a recent history of pilgrimage travel associated with prolonged exposure to crowded environments, representing a significant epidemiological risk factor, while the second case was a pediatric patient who demonstrated a rapid and favorable clinical response to targeted antimicrobial therapy. In the two FN cases with CSF cultures positive for *E. coli*, one neonate had a prior history of surgery for a benign neoplasm and showed clinical improvement with antimicrobial treatment followed by discharge, whereas the other patient had a predisposing condition of a ventriculoperitoneal shunt and presented with the rapid onset of fever, vomiting, and neck stiffness; this patient also responded promptly to appropriate antibiotic therapy.

**TABLE 3:** Characteristics of discordant results: false‐negative and false‐positive cases.

Discordant category	Panel result	Patient age	CSF culture	CSF WBC (cells/mm^3^)	CSF Gram stain	CSF protein concentration (mg/dL)	CSF glucose (mg/dL)	Physical examination findings∗	Clinical diagnosis (ME or non‐ME)	Outcome
FN	Neg	63	*S. pneumoniae*	50	Gram‐pos. cocci	137	78	Pos	ME	Death
FN	Neg	7	*S. pneumoniae*	80	Gram‐pos. cocci	79	61	Pos	ME	Discharge
FN	Neg	< 1	*E. coli*	30	Gram‐neg. bacilli	72	46	Neg	ME	Discharge
FN	Neg	5	*E. coli*	70	Gram‐neg. bacilli	39	85	Pos	ME	Discharge
FP	HHV‐6	71	Neg	0	Neg	37	179	Neg	Non‐ME	Discharge
FP	*H. influenzae*	11	Neg	0	Neg	27	60	Neg	Non‐ME	Discharge
FP	*Enterovirus*	32	Neg	0	Neg	78	43	Neg	non‐ME	Discharge
FP	*Enterovirus*	38	Neg	10	Neg	97	41	Neg	non‐ME	Discharge
FP	HHV‐6	39	Neg	0	Neg	129	53	Neg	non‐ME	Discharge

*Note:* CSF, cerebrospinal fluid; Neg, negative; Pos, positive.

Abbreviations: FN = false negative; FP = false positive.

^∗^Physical examination findings suggestive of meningoencephalitis.

A total of five FP results were identified, including one pediatric patient with a positive *H. influenzae* result on the panel despite the absence of growth in cerebrospinal fluid culture, normal microscopic and laboratory findings inconsistent with meningitis, and a final clinical diagnosis unrelated to meningitis with the primary presentation being epileptic seizures; in the remaining four cases interpreted as FP, *Enterovirus* was detected in two patients and HHV‐6 in two patients, although none of these individuals demonstrated clinical or laboratory features supportive of meningoencephalitis (Table 3). Among the FP cases with detection of HHV‐6, a 71‐year‐old male patient presented with gait and speech difficulties; however, physical examination findings were not indicative of meningoencephalitis, electroencephalography was inconsistent with encephalitis, and imaging and laboratory studies did not support an encephalitic process. The patient was discharged without antiviral therapy. Another patient, a 39‐year‐old male, presented with memory impairment, disorientation, and speech difficulties. Clinical, radiological, and laboratory findings were not suggestive of encephalitis, although brain MRI revealed patchy areas of hyperintense contrast uptake. HHV‐6 was not considered the causative agent in this case, and the patient’s symptoms resolved following corticosteroid therapy prior to discharge. A 32 year‐old male patient with detection of *Enterovirus* on the ME panel presented with diplopia, ptosis, and ocular pain. Clinical, laboratory, and imaging findings were not consistent with encephalitis. A diagnosis of Tolosa−Hunt syndrome was considered, and the patient’s symptoms improved following corticosteroid therapy. A 38‐year‐old male patient with detection of *Enterovirus* on the ME panel presented with occipital headache. Clinical, laboratory, and imaging findings were not consistent with encephalitis. A right internal carotid artery aneurysm was identified, and the patient underwent appropriate interventional procedures before discharge. In all of these patients with discordant results, cerebrospinal fluid PCR and culture results for *Mycobacterium tuberculosis* were also negative.

During the study period, bacterial pathogens not included in the panel were detected in 38 patients. The most frequently identified organisms were *Acinetobacter baumannii* (42.8%) and *Stenotrophomonas maltophilia* (20%), followed by *Enterococcus faecium* (5.7%), *Klebsiella pneumoniae* (5.7%), and *Enterobacter aerogenes* (5.7%), and others (Table 4). In addition, *M*. *tuberculosis* was detected in 3 patients. Overall, these findings indicate that, while the panel covers key bacterial agents, a substantial proportion of infections were caused by organisms not included in the panel (Table 4).

**TABLE 4:** Frequency and distribution of bacterial pathogens not included in the ME panel detected in patients during the study period.

Microorganism	*n* (%)
*Acinetobacter baumannii*	15 (42.8)
*Stenotrophomonas* *maltophilia*	7 (20)
*Enterococcus faecium*	2 (5.7)
*Klebsiella pneumoniae*	2 (5.7)
*Enterobacter aerogenes*	2 (5.7)
*Pseudomonas aeruginosa*	1 (2.8)
*Proteus mirabilis*	1 (2.8)
*Staphylococcus aureus*	1 (2.8)
*Salmonella spp*.	1 (2.8)
*Mycobacterium tuberculosis*	3 (8.5)

## 4. Discussion

In the present study, we evaluated the clinical performance and real‐world utility of the BioFire FilmArray ME panel in a large cohort of 955 unique patients, representing one of the largest single‐center CSF datasets reported to date. The overall positivity rate of 8.4% observed in our cohort is consistent with prior epidemiological studies. The identification of *S. pneumoniae* as the primary bacterial pathogen, with *H. influenzae* and *N. meningitidis* occurring at lower frequencies, corresponds closely with the recognized epidemiologic pattern observed in community‐acquired bacterial meningitis [16]. In parallel, *Enterovirus* and HSV‐1 represented the most commonly identified viral agents, a pattern that is consistent with prior epidemiologic reports [6, 17].

The ME panel showed an overall sensitivity of 94.8% and a specificity of 99.4% in this study, supporting its strong diagnostic utility under real‐world clinical conditions. These findings are consistent with the pre‐FDA prospective multicenter evaluation, which documented sensitivities greater than 95% for the majority of analytes and specificities surpassing 99% across all targets [6]. In addition, a meta‐analysis assessing ME panel performance found a pooled sensitivity of 90% and a specificity of 97%, further underscoring the reliability of multiplex molecular diagnostic platforms across varied clinical environments [9]. The slightly higher sensitivity observed in adults (97.6%) compared to pediatric patients (91.6%) in our cohort may reflect age‐related differences in pathogen burden, CSF cellular response, and disease severity. Also, pathogen distribution may vary according to patient age, and in infants, particularly, limited CSF sample volume and prior antibiotic exposure may reduce the diagnostic yield of culture. The FilmArray ME PCR panel identified *S. agalactiae* and *E. coli* in CSF samples from infants with suspected meningitis, detecting pathogens not only in culture‐positive cases but also in culture‐negative samples obtained after antibiotic exposure in the presence of CSF findings consistent with meningitis [18].

Our PPVs for bacterial (97.3%) and viral agents (90.0%) were high, although lower PPVs for selected viral targets were observed, consistent with prior reports. In the pre‐FDA evaluation, the PPV was substantially lower for several targets, including *E. coli* K1, *S. agalactiae*, *S. pneumoniae*, CMV, HSV‐1, and *C. neoformans*/*C. gattii* (6). Similarly, post‐FDA clearance of the ME panel, a retrospective analysis reported positive percent agreement values between 83% and 100% across the evaluated targets [17]. The fungal PPV in our cohort reached 100%; however, this estimate is limited by the only two positive fungal cases, emphasizing the need for cautious interpretation. Among the four FN results identified in our study, all were bacterial and involved *S. pneumoniae* or *E. coli*, pathogens that are included in the ME panel. Importantly, all FN patients exhibited clinical and laboratory findings consistent with meningitis, and CSF cultures were positive. Nevertheless, pooled evidence from the meta‐analysis showed that HSV‐1, HSV‐2, *Enterovirus,* and *C. neoformans/gattii* exhibited the highest rates of FN detections [9].

Five FP results were documented in our cohort, involving HHV‐6, *H. influenzae*, and *Enterovirus.* Notably, HHV‐6 was detected in two patients without supportive clinical or radiological evidence of encephalitis, consistent with prior studies reporting that a high proportion of HHV‐6–positive ME panel results may be clinically insignificant [19]. The interpretive challenge is heightened by the inherent capacity of the herpesviruses included in the ME panel (HSV‐1, HSV‐2, CMV, VZV, and HHV‐6) to enter a latent state. As a result, positive results may reflect latent viral genomes carried by CSF leukocytes or peripheral blood contamination from a traumatic lumbar puncture instead of genuine CNS involvement [20]. In addition, HHV‐6 may become integrated into host chromosomal DNA and transmitted vertically, producing sustained PCR detection that does not necessarily indicate clinical infection [21]. These biological features likely played a role in the discordant HHV‐6 findings identified in our study.

Isolation of a pathogen from CSF by culture remains the reference standard for confirming bacterial and fungal meningitis, whereas for viral etiologies, conventional real‐time PCR is widely accepted as the diagnostic gold standard [22, 23]. In addition to these methods, researchers may incorporate clinical findings, antigen testing, and CSF parameters to develop supportive diagnostic criteria and integrated diagnostic algorithms. Across studies, the ME panel has been associated with approximate FP and FN rates of 11.4% and 2.2%, respectively, when evaluated against conventional reference standard techniques [9]. Our data corroborate the established performance profile and reinforce the principle that ME panel findings should be interpreted alongside complementary clinical and laboratory information. The systematic evaluation of discordant cases demonstrates that molecular results, if not integrated with CSF analyses, neuroimaging, and clinical assessment, have the potential to misguide antiviral or antibacterial management. To enhance diagnostic accuracy in difficult scenarios, future prospective studies are warranted, particularly for circumstances involving traumatic lumbar puncture, differentiation of latent or reactivating viruses, and instances in which clinical suspicion is low or CSF indices remain within normal limits [19].

Our study also revealed that 38 patients harbored bacterial pathogens not represented in the ME panel, most frequently *A. baumannii* and *S. maltophilia*. These organisms accounted for a sizable fraction of CSF infections and are well recognized as causes of healthcare‐associated and neurosurgical infections rather than classic community‐acquired meningitis. Previous studies have similarly documented limitations in the panel’s pathogen spectrum, emphasizing that, despite its focus on the principal agents of community‐acquired meningitis, the ME panel should not be viewed as a substitute for conventional culture or broader diagnostic workups, particularly in individuals with implanted devices, neurosurgical interventions, or intensive care–related risks [23, 24].

The use of syndromic testing through the ME panel confers several meaningful advantages, including markedly faster diagnostic reporting, earlier initiation of targeted therapy, more timely discontinuation of unnecessary antimicrobial agents, and a reduced need for further investigative testing [25]. Despite these advantages, appropriate application of the ME panel necessitates robust diagnostic stewardship and the development of explicit testing frameworks created jointly by laboratory professionals and infectious disease clinicians [26]. Despite its expanding global use, best practice recommendations for integrating the ME panel into everyday clinical workflows are not yet well established. As a result, numerous laboratories encounter difficulty in defining rational restriction criteria in the absence of sufficient real‐world data [25]. This work is subject to important limitations. With respect to viral agents, the reference method used for comparison was not a definitive gold standard and included assays such as PCR. Another important limitation is the absence of a third arbiter method, such as next‐generation sequencing, for the resolution of discordant results between the ME panel and reference methods. The study relied exclusively on data from one center, thereby limiting broader applicability. The retrospective design introduces susceptibility to incomplete records and unrecognized confounders. Furthermore, the limited sample sizes within specific subgroups and the possibility of selection bias may have influenced the observed outcomes.

In conclusion, our large real‐world cohort demonstrates that the FilmArray ME panel is a highly sensitive and specific diagnostic tool for the rapid detection of major meningitis and encephalitis pathogens. However, the occurrence of FN bacterial results, the identification of viral targets with limited clinical significance, and the considerable proportion of infections caused by organisms not included in the panel collectively underscore that this assay should serve as an adjunct rather than a replacement for conventional microbiological methods and thorough clinical evaluation.

## Funding

This research received no external funding.

## Conflicts of Interest

The authors declare no conflicts of interest.

## Data Availability

The data that support the findings of this study are available on request from the corresponding author.
